# Protein complex detection in PPI networks based on data integration and supervised learning method

**DOI:** 10.1186/1471-2105-16-S12-S3

**Published:** 2015-08-25

**Authors:** Feng Ying Yu, Zhi Hao Yang, Xiao Hua Hu, Yuan Yuan Sun, Hong Fei Lin, Jian Wang

**Affiliations:** 1 College of Computer Science and Technology, Dalian University of Technology, Dalian, China; 2 College of Computing &Informatics, Drexel University, Philadelphia, PA 19104, USA

**Keywords:** Protein-protein interaction network, Protein complexes, Data integration, Supervised learning

## Abstract

**Background:**

Revealing protein complexes are important for understanding principles of cellular organization and function. High-throughput experimental techniques have produced a large amount of protein interactions, which makes it possible to predict protein complexes from protein-protein interaction (PPI) networks. However, the small amount of known physical interactions may limit protein complex detection.

**Methods:**

The new PPI networks are constructed by integrating PPI datasets with the large and readily available PPI data from biomedical literature, and then the less reliable PPI between two proteins are filtered out based on semantic similarity and topological similarity of the two proteins. Finally, the supervised learning protein complex detection (SLPC), which can make full use of the information of available known complexes, is applied to detect protein complex on the new PPI networks.

**Results:**

The experimental results of SLPC on two different categories yeast PPI networks demonstrate effectiveness of the approach: compared with the original PPI networks, the best average improvements of 4.76, 6.81 and 15.75 percentage units in the F-score, accuracy and maximum matching ratio (MMR) are achieved respectively; compared with the denoising PPI networks, the best average improvements of 3.91, 4.61 and 12.10 percentage units in the F-score, accuracy and MMR are achieved respectively; compared with ClusterONE, the start-of the-art complex detection method, on the denoising extended PPI networks, the average improvements of 26.02 and 22.40 percentage units in the F-score and MMR are achieved respectively.

**Conclusions:**

The experimental results show that the performances of SLPC have a large improvement through integration of new receivable PPI data from biomedical literature into original PPI networks and denoising PPI networks. In addition, our protein complexes detection method can achieve better performance than ClusterONE.

## Background

Protein-protein interactions (PPI) are fundamental to the biological processes within a cell. Beyond individual interactions, there is a lot more systematic information contained in protein interaction graphs. Complex formation is one of the typical patterns in this graph and many cellular functions are performed by these complexes containing multiple protein interaction partners. Many automatic approaches have been proposed to detect the protein complexes from PPI networks, such as CMC [1], COACH [2], MCODE [3], MCL [4], Cfinder [5], and ClusterONE [6]. However, most of these methods are based on unsupervised graph clustering methods and predict protein complexes only with pre-defined rules. Compared with them, supervised learning methods [7,8] can utilize the known complexes information and may achieve better performances.

At present, large number of PPI databases have been created. Gavin [9], Krogan [10] and DIP [11] are popular PPI databases used by the protein complex detection methods. However, these databases are sparse since the fraction of known true physical interactions is limited [12]. For example, the average numbers of interactions per protein are 6.98, 7.86, and 9.13 in DIP, Krogan, and Gavin, respectively. Nevertheless, large amounts of PPIs could be found in the rapidly growing biomedical literature. Furthermore, since these PPI data are provided by biomedical experts, they are relatively accurate. Their Integration with the existing PPI datasets can be hopeful to eliminate the PPI networks' sparsity, and, therefore, improve the complex detection performance.

In this paper, we present a complex detection approach based on data integration and supervised learning. In this approach, the new PPI networks are constructed by integrating PPI datasets with the PPI data extracted by PPIExtractor [13] from biomedical literature, and then the less reliable PPI between two proteins are filtered out based on semantic similarity and topological similarity of the two proteins. Finally, the supervised learning protein complex detection (SLPC) method, which can make full use of the information of available known complexes, is applied to detect protein complex on the new PPI networks. The experimental results demonstrate that our approach outperform ClusterONE, the state-of-the-art method.

## Methods

### Extracting PPI data with PPIExtractor

In our work, we use PPIExtractor [13] to extract PPI interactions from biomedical literature and then integrate them into the PPI networks. PPIExtractor is a useful tool publicly available for extracting new PPI data from a large collection of biomedical literature. Experimental evaluations show that it can achieve state-of-the-art performance on a DIP subset with respect to comparable evaluations.

**Figure 1:**
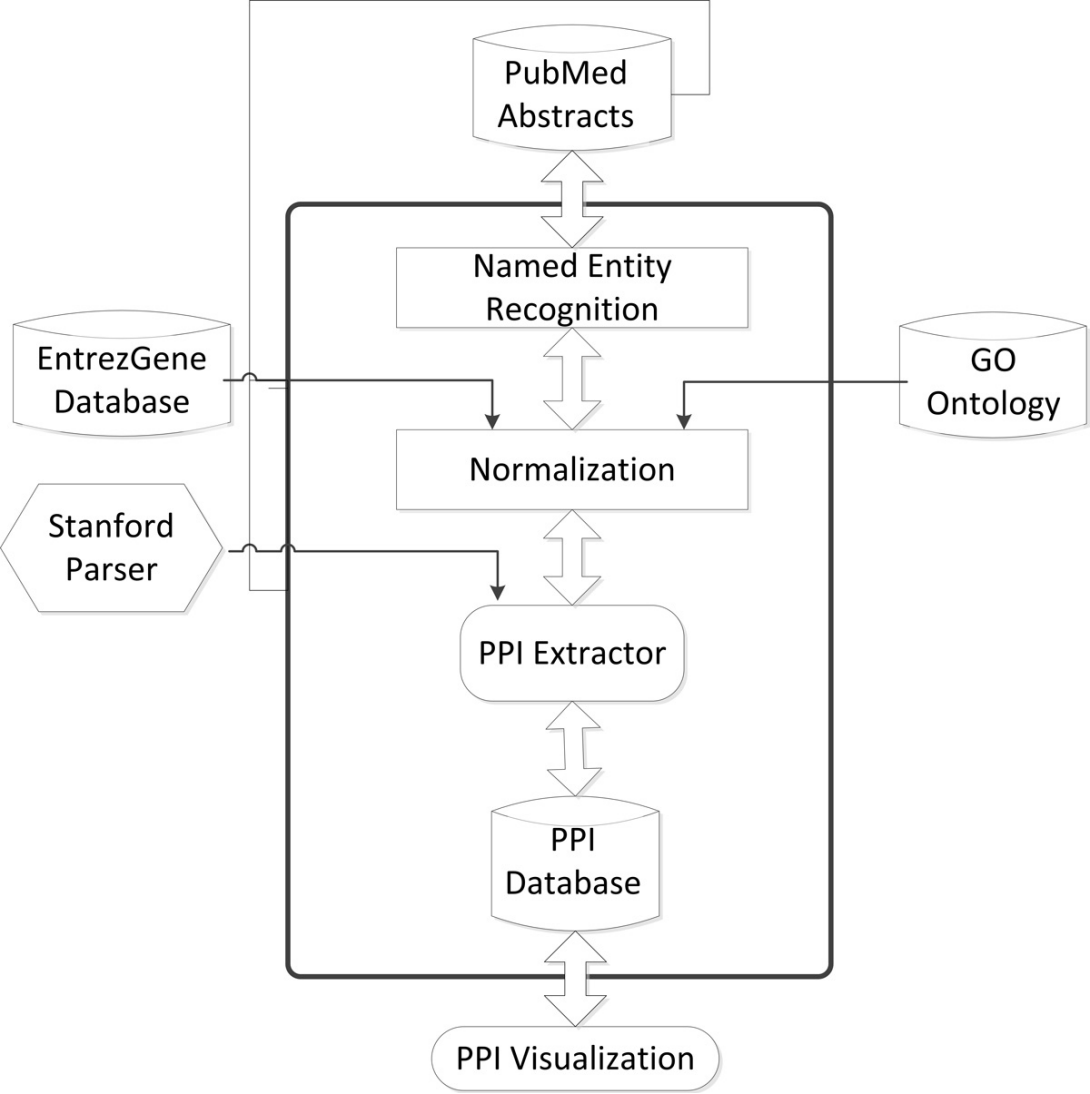
**The architecture of PPIExtractor**.

PPIExtractor contains four modules: (i) Named Entity Recognition (NER) module which aims to identify the protein names in the biomedical literature; (ii) Normalization module which determines the unique identifier of proteins identified in NER module; (iii) PPI extraction module which extracts the PPI information in the biomedical literature; (iv) PPI visualization module which displays the extracted PPI information in the form of a graph. Figure 1 shows the architecture of PPIExtractor.

127,217 PubMed abstracts were downloaded from PubMed website (http://www.ncbi.nlm.nih.gov/pubmed) with the query string "((Saccharomyces cerevisiae) OR yeast) AND protein" and PPIExtractor extracted a total of 126,165 protein interactions from these abstracts.

Since most of the protein names in the PPI databases are systematic names for nuclear-encoded ORFs begin with the letter 'Y' (for 'Yeast') while those in PubMed abstracts are not, we built a yeast protein alias name list with about 6,000 entries from the UniProt website(http://www.uniprot.org/uniprot/? query=yeast&sort= score). The list is used to convert the protein names in PubMed abstracts to systematic names for nuclear-encoded ORFs.

### PPI datasets

DIP, Krogan, Gavin, three yeast PPI datasets, are used in our work. The details of these PPI datasets are shown in Table 1. For each dataset, original PPI and denoising PPI networks are built, respectively, to verify our method's effectiveness. Original PPI networks are original three yeast PPI datasets mentioned above. Denoising PPI networks are three filtered PPI datasets, in which low reliability interactions are removed with different denoising thresholds. As a matter of fact, protein interaction data produced by high-throughput experiments are often associated with high false positive and false negative rates. Therefore, a method based on both semantic and topological similarity of the two proteins is applied in our work to measure the reliability of the interaction. GO (The Gene Ontology Consortium [14]) annotation from SGD [15] is used in this measurement approach. In this method, a PPI's reliability is defined as formula (1):

**Table 1:** Properties of three yeast PPI datasets.

Datasets	DIP	Krogan	Gavin
Number of proteins	4928	3581	1430
Number of interactions	17201	14076	6531

(1) $$rel\left(m,n\right)=\sqrt{-|C\left(m,n\right)|\times \text{log}\left(\frac{\text{min}|T_{i}\left(m,n\right)|}{T_{\text{max}}}\right)+NE\left(m,n\right)}$$

Where |*C*(*m, n*)| denotes the number of terms in *C*(*m, n*), the set of the GO terms in which annotation proteins *m *and *n *are included. | *T_i_*(*m, n*) | denotes the number of terms in *T_i_*(*m, n*), the set of annotated proteins on GO term *g_i _*in whose annotation *m *and *n *are included. *T_max _*denotes the maximum size of annotated proteins on all GO terms. The GO term's specificity can be quantified by the proportion of the annotation size of a GO term (*T_i_*(*m,n*)) to the total number of annotated proteins (*T_max_*), i.e. a GO term is regarded to be more specific if it has less annotated proteins. *NE*(*m, n*) denotes the number of neighbors that *m *and *n *share. The formula (1) demonstrates that if the GO term proteins *m *and *n *share is more specific, or if they have more common neighbors or GO terms, the interaction between them is more reliable. The details of the denoising PPI networks are shown in Table 2.

**Table 2:** Properties of denoising PPI networks with different denoising thresholds.

den_thred	DIP		Krogan		Gavin	
	
	**#Pro**.	**#Int**.	**#Pro**.	**#Int **.	**#Pro**.	**#Int**.
0.5	3669	11617	2400	9507	1318	5971
0.6	3536	11316	2331	9367	1315	5963
0.7	3536	11316	2331	9367	1315	5963
0.8	3490	11190	2309	9313	1312	5958
0.9	3450	11084	2273	9223	1304	5942
1.0	3402	10933	2235	9143	1301	5928
1.1	3205	9486	2103	7736	1267	5492
1.2	3164	9381	2078	7676	1261	5486
1.3	3095	9219	2043	7572	1255	5469
1.4	3011	9019	1998	7451	1252	5449

**#den_thred**. denotes the different denoising thresholds; **#Pro**. denotes the number of proteins. **#Int**. denotes the number of interactions.

### Integration of the extracted PPI data into the PPI networks

PPIExtractor assigns the extracted PPIs from the biomedical literature weights representing their reliability [13]. In our study, only PPIs with the weights equal to or higher than an integrating threshold are integrated into the original PPI dataset. In addition, both two proteins in a new PPI should already exist in the PPI dataset. The amounts of the PPI added into the original PPI networks with different integrating thresholds are shown in Table 3.

**Table 3:** The amounts of the PPIs added into the original PPI networks with different integrating thresholds.

int_thred	DIP	Krogan	Gavin
0	1206	857	205
-0.1	1661	1166	288
-0.2	2197	1525	371
-0.3	2789	1953	455
-0.4	3534	2470	568
-0.5	4470	3079	684
-0.6	5713	3907	866
-0.7	7257	4879	1096
-0.8	9153	6091	1447
-0.9	11314	7482	1821
-1.0	13257	8669	2125
-1.1	14241	9242	2270
-1.2	14580	9433	2315

**#int_thred**. denotes the different integrating thresholds.

The weights of the PPIs added into the denoising PPI networks are higher than the integrating threshold -0.6. the reason is that our SLPC method have the best performance on the original PPI networks with the integrating threshold -0.6. What is more, the PPIs, when integrated into the denoising PPI networks, are also filtered with different denoising thresholds. The amounts of the PPIs added with different denoising thresholds are shown in Table 4.

**Table 4:** The amounts of the PPIs added into the denoising PPI networks with different denoising thresholds.

den_thred	DIP	Krogan	Gavin
0.5	4214	2149	685
0.6	4156	2110	684
0.7	4156	2110	684
0.8	4132	2103	683
0.9	4088	2069	679
1.0	4062	2032	678
1.1	3411	1612	590
1.2	3357	1595	585
1.3	3277	1546	581
1.4	3204	1524	570

**#den_thred**. denotes the different denoising thresholds.

### Protein complexes detection with SLPC

In our work, a supervised learning protein complex detection (SLPC) method is employed to predict the protein complexes from PPI networks. Currently, most of protein complex detection methods are unsupervised ones, without utilizing the known complexes information. However, in the research field of protein complexes, numerous complexes have been provided, which can be used as the prior knowledge of the complex detection methods. In previous work, we presented a supervised learning protein complex detection (SLPC) method to predict protein complexes [8]. The SLPC method utilizes the features including Graph density [3], Degree statistics, Edge weight statistics, Clustering coefficient [16], and Topologic change [17]. Experimental evaluations show that SLPC can achieve better performances than other present protein complex detection methods. SLPC algorithm is showed in Table 5 and more details are provided in [8].

**Table 5:** Protein complex detection algorithm.

**Input **: an unweighted network, a weighted network built via GO annotation and a training set Complex detection process: **Step 1**: construct the feature vector space for the complexes in the training set from the unweighted and weighted PIN networks and train the Regression model **Step 2**: find maximal cliques in the PIN by the Cliques algorithm -rank the clique set C={C_1_, C_2_, ..., C_n_} in descending order of the scores given by the Regression model -for each clique C_i_, check all the cliques (denoted as C_j_) with lower scores, if C_i_∩C_j _ > threshold, then remove C_j_. -output: the updated clique set **Step 3**: grow the cliques -for each clique C_i_, the set of its neighbors is denoted as N(C_i_), do update operation as follows: -check all the nodes in N(C_i_) -select v_i_∈N(C_i_), which makes v_i_∪C_i _achieve higher score given by the Regression model -update C_i_= v_i_∪C_i_, N(C_i_) = N(C_i_) - v_i_ -repeat the update operation until there is no node v_j _in N(C_i_) that leads to score(v_j_∪C_i_) > score(C_i_) -output: the candidate complex set C = {C_1_, C_2_, ..., C_n_} **Step 4**: filter the candidate complexes -rank the candidate complexes in descending order of the score given by the Regression model -for each candidate complex C_i_, check all the candidates C_j _with lower scores -if overlap (C_i_, C_j_) > merg_thred if score(C_i_∪C_j_) > score(C_i_) do merge operation: update C_i _= C_i_∪C_j_ else do remove operation: remove C_j _from the candidate set **output**: the predicted complex set	

## Experiments and results

### Gold standard protein complexes

We constructed the gold standard protein complexes by combining MIPS [18], Aloy [19], SGD [15] with TAP06 [9]. Proteins absent from the corresponding PPI networks are filtered out from the gold standard. In addition, only the protein complexes including at least two different proteins are retained as the research shows that most of the protein complexes include more than one protein [20]. The details of the gold standard protein complexes of original PPI networks and denoising PPI networks are shown in Tables 6 and 7, respectively.

**Table 6:** The details of the gold standard protein complexes of original PPI networks.

	DIP	Krogan	Gavin
Number of complexes	732	623	584
The average size of complexes	7.18	6.95	6.27

**Table 7:** The details of the gold standard protein complexes of denoising PPI networks with different denoising thresholds.

den_thred	DIP		Krogan		Gavin	
	
	#complex	#size	#complex	#size	#complex	#size
0.5	679	7.01	565	7.04	542	6.49
0.6	673	7.03	563	7.03	542	6.49
0.7	673	7.03	563	7.03	542	6.49
0.8	673	7.01	563	7.02	542	6.49
0.9	668	7.03	557	7.03	534	6.55
1.0	667	7.03	552	7.07	533	6.56
1.1	660	7.03	541	7.1	518	6.66
1.2	658	7.04	539	7.12	517	6.67
1.3	653	7.06	538	7.11	517	6.67
1.4	649	7.05	533	7.12	515	6.68

**#den_thred**. denotes the different denoising thresholds; **#Den**. denotes the denoising PPI network; **# Den.Ext**. denotes the denoising extended PPI network (denoising PPI network added new PPI data); **#complex **denotes the number of complexes; **#size **denotes the average size of complexes.

### Evaluation metrics

In our study, F-score, Accuracy (Acc), maximum matching ratio (MMR) are used as the evaluation metrics. The neighborhood affinity score NA(A, B) defined as follows is used to evaluate the similarity of two protein complexes A and B:

$$NA\left(A,B\right)=\frac{|VA\cap VB{|}^{2}}{\left|VA|\times |VB\right|}$$

If the NA(A, B) is large than or equal to 0.25, complexes A and B are regarded to be matching.

F-score, a popular metric of evaluating complex detection method, is used as the first measure to evaluate the performance.

(3) $$N_{cb}=|\left\{b|b\in B,\exists p\in P,NA\left(p,b\right)\geq 0.25\right\}|$$

(4) $$N_{cp}=|\left\{p|p\in P,\exists b\in B,NA\left(p,b\right)\geq 0.25\right\}|$$

(5) $$\text{Pr}ecision=\frac{N_{cp}}{\left|P\right|},\text{Re}call=\frac{N_{cb}}{\left|B\right|}$$

(6) $$F-score=\frac{2\times \text{Pr}ecision\times \text{Re}call}{\left|\text{Pr}ecision+\text{Re}call\right|}$$

Where *P *and *B *are the predicted and gold standard complex sets, respectively; *Ncb *is the number of the gold standard complexes matching at least one predicted complex and *Ncp *is the number of the predicted complexes matching at least one gold standard complex and F-score is calculated as the harmonic mean of precision and recall values.

The second measure we used is the geometric accuracy as introduced by Broh´ee et al. [21], which is the geometric mean of clustering-wise sensitivity (*Sn*) and clustering-wise positive predictive value (*PPV*). A high *Sn *value indicates that the protein complex prediction has a good coverage of the proteins in the gold standard complexes, and a high *PPV *value indicates that the predicted protein complexes are likely to be true protein complexes. Assuming the number of the gold standard complexes is *n *and the number of the predicted complexes is m. *T_ij _*denotes the number of proteins that are found both in gold standard complex *i *and predicted complex *j*. The *Sn, PPV, Acc *are defined as follows:

$$Sn=\frac{\sum_{i=1}^{n}\underset{j}{\text{max}}\left\{T_{ij}\right\}}{\sum_{i=1}^{n}N_{i}}$$

$$PPV=\frac{\sum_{j=1}^{m}\underset{i}{\text{max}}\left\{T_{ij}\right\}}{\sum_{i=1}^{m}T_{.j}}$$

$$T_{.j}=\sum_{i=1}^{n}T_{ij}$$

$$Acc=\sqrt{Sn\times PPV}$$

The third metric we used is the maximum matching ratio (MMR) [6], which is based on a maximal one-to-one mapping between gold standard complex and predicted complex.

$$MMR=\frac{\sum_{i=1}^{n}\underset{j}{\text{max}}NA\left(i,j\right)}{n}$$

Where *n *denotes the number of the gold standard complexes; *m *the number of the predicted complexes; *j *as the member of the predicted complexes. MMR offers a natural, intuitive way to compare predicted complexes with a gold standard and it explicitly penalizes cases when a reference complex is split into two or more parts in the predicted set, as only one of its parts is allowed to match the correct reference [6].

The Acc measure explicitly penalizes predicted complexes that do not match any of the reference complexes. However, gold standard sets of protein complexes are often incomplete [22]. As a consequence, predicted complexes not matching any known reference complexes may still exhibit high functional similarity or be highly co-localized, and therefore they could still be prospective candidates for further in-depth analysis. In other words, a predicted complex that does not match a reference complex is not necessarily an undesired result, and optimizing for the geometric accuracy measure might prevent us from detecting novel complexes from a PPI dataset. Therefore, in the performance comparison, the F-score and MMR are used as the main metrics; the Acc is only used as an auxiliary one.

### The performances of SLPC on original PPI networks

First we tested SLPC on three original PPI networks, i.e. DIP, Krogan and Gavin. The results of F-score, accuracy and MMR are shown in Tables 8, 9, and 10, respectively. It can be seen that the performances measured with these metrics keep improving on these networks with the integrating threshold decreasing from 0 to -0.6. With the threshold -0.6, SLPC achieves the highest average improvements on all three original PPI networks: 4.76, 6.81 and 15.75 percentage units in F-score, accuracy and MMR, respectively. This shows that the introduction of PPIs extracted from literature into the original PPI datasets can boost the performance. The reason is that, the higher integrating threshold means more reliable new PPI interactions are integrated into the original PPI networks, which relieves the sparse problem of PPI networks. As shown in Table 11, in most cases, the average size of complexes predicted from extended PPI networks is much closer to the one of the gold standard protein complexes than that from the original PPI networks, and, therefore, SLPC achieves better performance on extended PPI networks than on original PPI networks.

**Table 8:** The F-score performances of SLPC on original PPI networks with different integrating thresholds.

int_thred	DIP	Krogan	Gavin	Avg.Δ
Origin	0.5531	0.5029	0.6389	
0	0.5543	0.5298	0.6518	2.53%
-0.1	0.5463	0.5348	0.6665	3.14%
-0.2	0.5481	0.5382	0.6658	3.44%
-0.3	0.5621	0.5515	0.6623	4.98%
-0.4	0.5527	0.5485	0.6642	4.32%
-0.5	0.5577	0.544	0.6638	4.30%
**-0.6**	**0.553**	**0.5543**	**0.665**	**4.76%**
-0.7	0.5418	0.5355	0.6638	2.78%
-0.8	0.5409	0.5329	0.6685	2.80%
-0.9	0.5335	0.5471	0.6694	3.34%
-1.0	0.5224	0.5445	0.6511	1.54%
-1.1	0.5138	0.5403	0.6501	0.69%
-1.2	0.5166	0.5368	0.6487	0.56%

**#int_thred**. denotes the different integrating thresholds; **Avg.Δ **denotes the average F-score improvement over that on the original PPI networks.

**Table 9:** The Accuracy performances of SLPC on original PPI networks with different integrating thresholds.

int_thred	DIP	Krogan	Gavin	Avg.Δ
Origin	0.3212	0.2984	0.3238	
0	0.323	0.3112	0.3285	2.10%
-0.1	0.3249	0.3108	0.3309	2.50%
-0.2	0.3241	0.3185	0.3331	3.50%
-0.3	0.3275	0.3233	0.3327	4.35%
-0.4	0.3284	0.3249	0.3336	4.72%
-0.5	0.3339	0.3301	0.3336	5.87%
**-0.6**	**0.3353**	**0.3347**	**0.3363**	**6.81%**
-0.7	0.3401	0.337	0.3369	7.62%
-0.8	0.3424	0.3411	0.3383	8.46%
-0.9	0.3397	0.3409	0.3367	8.00%
-1.0	0.3453	0.3428	0.3376	8.88%
-1.1	0.3423	0.3425	0.3378	8.56%
-1.2	0.3427	0.342	0.3386	8.63%

**Table 10:** The MMR performances of SLPC on original PPI networks with different integrating thresholds.

int_thred	DIP	Krogan	Gavin	Avg.Δ
Origin	0.306	0.2933	0.3562	
0	0.3156	0.3135	0.3646	4.13%
-0.1	0.3224	0.3180	0.3722	6.09%
-0.2	0.3269	0.3244	0.3802	8.06%
-0.3	0.3364	0.3328	0.3811	10.13%
-0.4	0.3385	0.3420	0.3880	12.05%
-0.5	0.3468	0.3529	0.3898	14.36%
**-0.6**	**0.3475**	**0.3600**	**0.3952**	**15.75%**
-0.7	0.3478	0.3603	0.3984	16.12%
-0.8	0.3603	0.3684	0.4000	18.55%
-0.9	0.3669	0.3767	0.4084	21.00%
-1.0	0.3626	0.3796	0.4064	20.67%
-1.1	0.3632	0.3767	0.4062	20.39%
-1.2	0.3633	0.3766	0.4087	20.62%

**Table 11:** The details of predicted complexes of SLPC on original PPI networks with different integrating thresholds.

int_ thred		DIP				Krogan				Gavin		
	
	#gl_sz	#cluster	#size	#matched	#gl_sz	#cluster	#size	#matched	#gl_sz	#cluster	#size	#matched
Origin	7.18	844	9.49	543	6.95	710	14.97	419	6.27	337	9.01	273
0	7.18	981	9.14	606	6.95	787	12.30	486	6.27	351	8.99	289
-0.1	7.18	1033	9.92	616	6.95	816	12.71	497	6.27	360	8.98	299
-0.2	7.18	1110	8.66	652	6.95	853	11.42	516	6.27	369	8.85	303
-0.3	7.18	1195	8.5	717	6.95	891	10.28	549	6.27	380	8.81	308
-0.4	7.18	1271	8.39	736	6.95	968	10.45	578	6.27	396	8.86	319
-0.5	7.18	1396	8.26	797	6.95	1040	10.03	595	6.27	397	8.94	318
-0.6	7.18	1580	8.42	889	6.95	1149	9.55	653	6.27	426	8.97	337
-0.7	7.18	1713	8.47	918	6.95	1243	9.26	663	6.27	446	9.07	347
-0.8	7.18	1928	8.72	1006	6.95	1402	9.24	718	6.27	475	8.89	367
-0.9	7.18	2147	8.77	1084	6.95	1555	9.10	816	6.27	521	9.01	395
-1.0	7.18	2171	9.12	1043	6.95	1612	8.83	834	6.27	540	8.88	393
-1.1	7.18	2139	9.40	995	6.95	1618	9.08	825	6.27	555	8.81	404
-1.2	7.18	2171	9.46	1016	6.95	1636	9.26	830	6.27	555	8.93	402

#**gl_size **denotes the average size of the gold standard protein complexes on original PPI networks; #**size **denotes the average size of predicted complexes. **#cluster **denotes the number of predicted complexes on extended PPI networks; **#matched **denotes the matching number between the predicted complexes and the gold standard protein complexes;

However, Tables 8 and 10 show that, F-score and MMR values begin to decline after they reach the highest values. The reason is that the lower integrating threshold will introduce more unreliable PPI interactions and therefore, deteriorate the performance of SLPC algorithm.

### The performances of SLPC on denoising PPI networks

Denoising PPI networks are the ones form which the low reliable PPIs are removed as discussed in the Section *PPI datasets*. And the denoising extended PPI networks are the ones into which the PPIs extracted from literature are integrated. More specifically, the new PPIs are also filtered out with different denoising thresholds like those PPIs in original PPI networks, and then integrated into the corresponding denoising PPI networks.

The performances of SLPC on denoising PPI networks are shown in Tables 12, 13 and 14. The performance of SLPC on the denoising extended PPI network is better than that on the corresponding denoising PPI network with any denoising threshold. With denoising threshold 0.9, SLPC achieves highest average improvement of 3.91, 4.61 and 12.10 percentage units in F-score, accuracy and MMR, respectively on denoising extended PPI networks over denoising PPI networks. This shows, once again, that the introduction the PPIs extracted from literature can boot the performance of complex detection methods.

**Table 12:** The F-score performances of SLPC on denoising PPI networks with different denoising thresholds.

Threshold	DIP		Krogan		Gavin		Avg.Δ
	
	**#Den**.	**#Den.Ext**.	**#Den**.	**#Den.Ext**.	**#Den**.	#Den.Ext	
0.5	0.5815	0.5889	0.5393	0.5761	0.6789	0.7006	3.76%
0.6	0.5849	0.5912	0.543	0.5854	0.6789	0.7021	4.10%
0.7	0.586	0.5905	0.5418	0.5778	0.6789	0.7012	3.57%
0.8	0.5834	0.5939	0.5414	0.5778	0.6767	0.7001	3.99%
0.9	0.5852	0.5962	0.5456	0.5819	0.6839	0.7057	3.91%
1.0	0.5881	0.596	0.5503	0.5864	0.6855	0.7072	3.69%
1.1	0.5538	0.5785	0.5624	0.5993	0.6627	0.7006	5.58%
1.2	0.5568	0.5776	0.5645	0.5972	0.6634	0.7015	5.09%
1.3	0.5572	0.582	0.5691	0.5984	0.6634	0.7011	5.09%
1.4	0.5537	0.5845	0.565	0.5989	0.6672	0.7065	5.82%
ClusterONE(0.9)	0.4412	0.4241	0.4834	0.4847	0.6418	0.6710	0.31%
Δ(0.9)		40.58%		20.05%		17.42%	26.02%

#Den. denotes the denoising PPI network. #Den.Ext. denotes the denoising extended PPI network. Avg.Δ denotes the average F-score improvement with the different denoising threshold over that on the corresponding denoising networks. Δ(0.9) denotes the improvement of SLPC over ClusterONE with the denoising threshold 0.9.

**Table 13:** The Accuracy performances of SLPC on denoising PPI networks with different denoising thresholds.

den_thred	DIP		Krogan		Gavin		Avg.Δ
	
	**#Den**.	**#Den.Ext**.	**#Den**.		**#Den**.	#Den.Ext	
0.5	0.331	0.3515	0.3215	0.3375	0.3309	0.3415	4.79%
0.6	0.3312	0.3508	0.322	0.3374	0.3312	0.3415	4.60%
0.7	0.3313	0.351	0.3216	0.3373	0.3312	0.3413	4.63%
0.8	0.3307	0.3513	0.3227	0.338	0.3308	0.3425	4.84%
0.9	0.3315	0.3516	0.3242	0.3393	0.3316	0.3419	4.61%
1.0	0.3314	0.3528	0.3256	0.3401	0.3324	0.3419	4.59%
1.1	0.3229	0.3482	0.3215	0.3391	0.3268	0.3412	5.91%
1.2	0.324	0.3478	0.3218	0.3401	0.327	0.3408	5.75%
1.3	0.3232	0.3477	0.3229	0.3405	0.327	0.3413	5.80%
1.4	0.3227	0.3461	0.3231	0.3397	0.327	0.34	5.45%
Clusterone(0.9)	0.4284	0.4267	0.3937	0.3985	0.4108	0.4124	0.40%
Δ(0.9)		-17.60%		-14.86%		-17.10%	-16.52%

**Table 14:** The MMR performances of SLPC on denoising PPI networks with different denoising thresholds.

den_thred	DIP		Krogan		Gavin		Avg.Δ
	
	#Den.	#Den.Ext.	#Den.		#Den.	#Den.Ext	
0.5	0.3294	0.3678	0.327	0.3817	0.3873	0.4225	12.49%
0.6	0.3319	0.3702	0.3281	0.3822	0.3879	0.4231	12.37%
0.7	0.3319	0.3703	0.3279	0.3816	0.3879	0.423	12.33%
0.8	0.3321	0.3709	0.3278	0.3813	0.3873	0.4224	12.36%
0.9	0.3342	0.3727	0.3317	0.3848	0.3935	0.428	12.10%
1.0	0.3347	0.3729	0.3351	0.3871	0.3943	0.4291	11.92%
1.1	0.3148	0.3565	0.3215	0.3789	0.3693	0.4098	14.02%
1.2	0.3151	0.3573	0.3203	0.3786	0.3703	0.4107	14.17%
1.3	0.3171	0.3585	0.3231	0.3797	0.3703	0.4098	13.75%
1.4	0.3154	0.3614	0.3247	0.382	0.3705	0.4125	14.52%
Clusterone(0.9)	0.2913	0.2829	0.3050	0.3188	0.3649	0.3730	1.29%
Δ(0.9)		31.74%		20.70%		14.75%	22.40%

In addition, Tables 12, 13 and 14 also show that, since the higher denoising threshold means more PPIs are filtered from the original PPI networks, which may lead to the missing of some real PPIs, the performances of SLPC algorithm on the denoising PPI networks and denoising extended PPI networks begin to decline after they reach the highest values.

The performance of ClusterONE, the state-of-the-art complex detection method, is also tested (its parameters are set as those described in [6]). With the denoising threshold 0.9, it achieves average improvements of 0.31, 0.40 and 1.29 percentage units in F-score, accuracy and MMR, respectively on denoising extended PPI networks over denoising PPI networks. This indicates that the introduction the PPIs extracted from literature can also boot the performance of ClusterONE. In addition, experimental results show that SLPC achieves better performance than ClusterONE. With the denoising threshold 0.9, the average performance improvement of SLPC over ClusterONE is 26.02 and 22.40 percentage units in F-score and MMR, respectively.

## Conclusions

Protein complexes, consisting of molecular aggregations of proteins assembled by multiple protein interactions, are of the fundamental units of macro-molecular organizations and play crucial roles in integrating individual gene products to perform useful cellular functions. Large amounts of PPI data generated by high-throughput experimental techniques can be used to predict protein complexes from PPI networks. At the same time, numerous accurate PPIs could be found in the rapidly growing biomedical literature since they are provided by biomedical experts. Their Integration with the existing PPI datasets can be hopeful to eliminate the PPI networks' sparsity, and, therefore, improve the complex detection performance.

In this paper, an approach of introducing PPIs from biomedical literature into existing PPI networks and applying supervised learning method in protein complex detection is presented. In the approach, the new PPI networks are constructed by integrating PPI datasets with the large and readily available PPI data from biomedical literature, and then the less reliable PPI between two proteins are filtered out based on semantic similarity and topological similarity of the two proteins. Finally, the supervised learning protein complex detection, SLPC, which can make full use of the information of available known complexes, is applied to detect protein complex on the new PPI networks.

The best average improvements of 4.76, 6.81 and 15.75 percentage units in F-score, accuracy and MMR are achieved respectively, on original extended PPI networks. In addition, the best average improvements of 3.91, 4.61 and 12.10 percentage units in F-score, accuracy and MMR are achieved, respectively, on denoising extended PPI networks. All these results show that, the introduction of PPIs extracted from literature into the original PPI datasets can boost the performance significantly. The reason is that the sparsity problem of PPI networks is remitted by integrating PPI data from biomedical literature. The results also show that our method outperforms ClusterONE, the state-of-the-art method. This is because our method makes full use of the information of available known complexes. To summarize, our complex detection method, based on supervised learning method and integrating PPI data from biomedical literature, can achieve the better performances than other complex detection methods.

## Competing interests

The authors declare that they have no competing interests.

## Authors' contributions

ZHY and FYY conceived of the study, carried out its design and drafted the manuscript. FYY performed the experiments. FYY, XHH, HFL, and JW participated in its design and coordination, and helped to draft the manuscript. All authors read and approved the final manuscript.

## Declarations

Publication of this article was funded by the following grants: the Natural Science Foundation of China (grant no. 61070098, 61272373 and 61340020), Trans-Century Training Programme Foundation for the Talents by the Ministry of Education of China (grant no. NCET-13-0084) and the Fundamental Research Funds for the Central Universities (grant no. DUT13JB09 and DUT14YQ213).

This article has been published as part of *BMC Bioinformatics *Volume 16 Supplement 12, 2015: Selected articles from the IEE International Conference on Bioinformatics and Biomedicine (BIBM 2014): Bioinformatics. The full contents of the supplement are available online at http://www.biomedcentral.com/bmcbioinformatics/supplements/16/S12.
